# Human activity’s impact on urban vegetation in China during the COVID-19 lockdown: An atypical anthropogenic disturbance

**DOI:** 10.1016/j.isci.2025.112195

**Published:** 2025-03-11

**Authors:** Yujie Li, Shaodong Huang, Panfei Fang, Yuying Liang, Jia Wang

**Affiliations:** 1 Beijing Key Laboratory of Precision Forestry, Beijing 100083, China; 2 State Key Laboratory of Efficient Production of Forest Resource, Beijing 100083, China

**Keywords:** Atmospheric science, Environmental science, Pollution, Ecology, Plants

## Abstract

The COVID-19 lockdown led to reduced industrial and transportation emissions in Chinese cities, improving air quality and affecting large-scale vegetation. This study examines changes in net primary productivity (NPP) across 283 prefecture-level cities in China (PCC) during the lockdown, focusing on aerosol optical depth (AOD), nighttime light (NTL), temperature, and precipitation. Results from spring 2020 show that 53.5% of cities experienced increased NPP, with greater gains in cities with high industrial and traffic activity due to reduced AOD. Structural equation modeling revealed that urban characteristics, particularly industrial levels, influenced NPP primarily through changes in AOD, with human activity shifts playing a larger role than climate factors. In cities with substantial NPP changes, human activity effects were especially pronounced. These findings highlight the complex interactions among urban characteristics, environmental changes, and vegetation responses, offering insights for ecological management and urban planning in the face of future disruptions.

## Highlights

• COVID-19 lockdown improved air quality and boosted urban vegetation in China
• NPP increased in 53.5% of Chinese cities during the lockdown
• Industrial and traffic-heavy cities showed greater NPP increases and AOD reductions
• Human activity changes had a stronger impact on NPP than climate factors

## Graphical abstract

**Figure:**
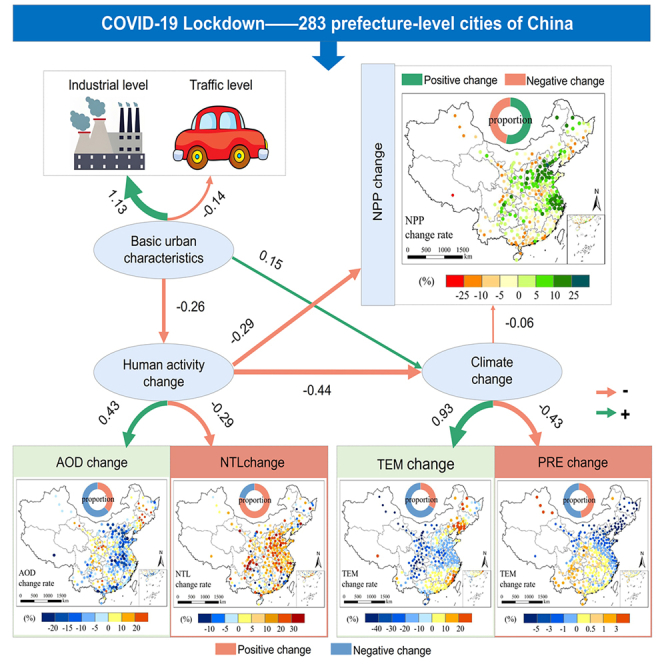

## Introduction

In early 2020, the COVID-19 pandemic rapidly spread worldwide, prompting governments to implement lockdown measures. These included closing borders, restricting movement, halting public transportation, and ceasing business and production activities.^1^ As a result, the gross domestic product (GDP) fell by 5.3% year-on-year in the first quarter of 2020.^2^ Despite the significant societal and economic impacts,^3^ the lockdowns had a relatively positive effect on the ecological environment.^4^ Numerous studies have confirmed the positive response of vegetation to this anthropogenic disturbance. Global vegetation productivity increased by 1.95% in 2020.^5^ In China, greener vegetation increased by 17.45%, as seen in the leaf area index (LAI) and gross primary productivity (GPP), with net primary productivity (NPP) in urban areas rising by 6.50% in spring 2020 compared to the average of 2017–2019.^6^^,^^7^ There was also a notable rise in the enhanced vegetation index (EVI) for crops, particularly in northern China.^8^ Similar trends were reported in India, where indices such as the normalized difference vegetation index (NDVI), solar-induced fluorescence (SIF), and EVI significantly increased during lockdowns.^4^^,^^9^ These changes in vegetation indices collectively point to a general improvement in vegetation growth. While these studies mainly focus on global or national vegetation shifts, there is a need to explore vegetation changes at the urban level, given the close link between cities and pandemic lockdowns.

Another significant impact of the COVID-19 lockdown was the dramatic reduction in human activities, particularly in urban areas, with work stoppages, production halts, and transportation limitations. China’s urbanization process has accelerated in recent years, leading to a sharp increase in urban populations and traffic volumes. This, in turn, has caused severe environmental pollution and heavy smog in cities.^10^^,^^11^^,^^12^^,^^13^ While during the COVID-19 pandemic, strict lockdowns severely impacted global industries.^14^ In China, industrial added value decreased by 8.5% compared to the previous year,^2^ while emissions fell by 10% year-on-year.^15^ Furthermore, travel restrictions reduced transportation by about 70%, with near-complete halts during the Spring Festival period.^16^^,^^17^^,^^18^ Statistics show a large-scale reduction in short-term human activities across China during COVID-19, accompanied by a marked improvement in air quality. Sulfur dioxide (SO_2_) and particulate matter (PM2.5) concentrations dropping significantly, and the average air quality index (AQI) decreasing by 7.8% in 44 northern cities.^18^^,^^19^

The intensity of this atypical anthropogenic disturbance needs to be quantified using appropriate indicators. Aerosol optical depth (AOD) is an important measure of urban air quality.^20^ It is mainly influenced by emissions from natural events such as wind and sandstorms, as well as human activities like transportation, thermal power generation, and industrial processes.^21^^,^^22^ Industrial production and vehicle exhaust emissions increase AOD by directly releasing particulate matter and generating secondary particles in the atmosphere.^23^ Since the main sources of AOD were significantly impacted by lockdown measures,^24^ changes in AOD before and after COVID-19 provide a clear reflection of air quality variations during this period. Additionally, nighttime light (NTL) is a direct indicator of human activity intensity,^25^ with urban lighting, industrial activities, and transportation networks being the main sources of NTL.^25^^,^^26^^,^^27^ The short-term changes in these human activities during the lockdown period (especially in urban areas) directly led to changes in NTL. Therefore, due to the restrictions on urban industry and transportation during the lockdown period, AOD and NTL serve as sensitive indicators to quantify the intensity of human disturbance. Studies have already confirmed the changes in both during COVID-19. China’s AOD declined by 6.8% overall and by 3.64% in urban areas in the spring of 2020.^6^^,^^28^^,^^29^ NTL exhibited a trend of first decreasing and then recovering.^30^

Human activities closely influence vegetation growth in urban areas.^31^ For AOD, on one hand, it affects photosynthesis by influencing direct or through diffuse radiation fertilization,^32^ impacting vegetation growth.^33^^,^^34^^,^^35^ On the other hand, lower air pollution reduces physical harm to plant leaves,^36^ enhancing photosynthetic activity and improving vegetation productivity.^37^ Also there is a correlation between NTL and vegetation growth. Some studies have proposed a link between NTL, NDVI, and vegetation productivity.^38^^,^^39^ Additionally, temperature (TEM) and precipitation (PRE), as key climatic parameters, directly influence vegetation growth through their variations.^40^ In summary, the changes in urban vegetation productivity during the lockdown period are a complex process influenced by both anthropogenic and natural disturbances.^41^^,^^42^ There is still a research gap in the interaction pathways and intensity of this process. In addition, there are varying levels of industry and transportation across different Chinese cities, so the intensity of anthropogenic disturbance caused by lockdown measures differs among cities. Whether this results in heterogeneous vegetation changes warrants further investigation.

To conclude, this study focuses on the prefecture-level cities of China (PCC), comparing vegetation NPP changes in spring (March to May) during the lockdown period (2020) with a reference period (2017–2019). We integrate statistical data to investigate the connection between industrial and traffic levels and NPP changes. Additionally, we construct a driver analysis model of NPP changes based on urban characteristics and environmental variables (AOD, NTL, TEM, and PRE) to explore the causes of vegetation changes during COVID-19. This study demonstrates how NPP varied at the urban scale in China throughout the pandemic, emphasizing the relationship with urban industrial and traffic levels. It clarifies the factors determining urban NPP changes during the lockdown period, highlighting the interdependence of urban characteristics, human activity, climate, and the ecological environment. These results offer strategies for vegetation conservation policies tailored to various types and scales of cities, essential for preserving urban ecosystems.

## Results

### Spatiotemporal changes in vegetation productivity in PCC

**Figure 1:**
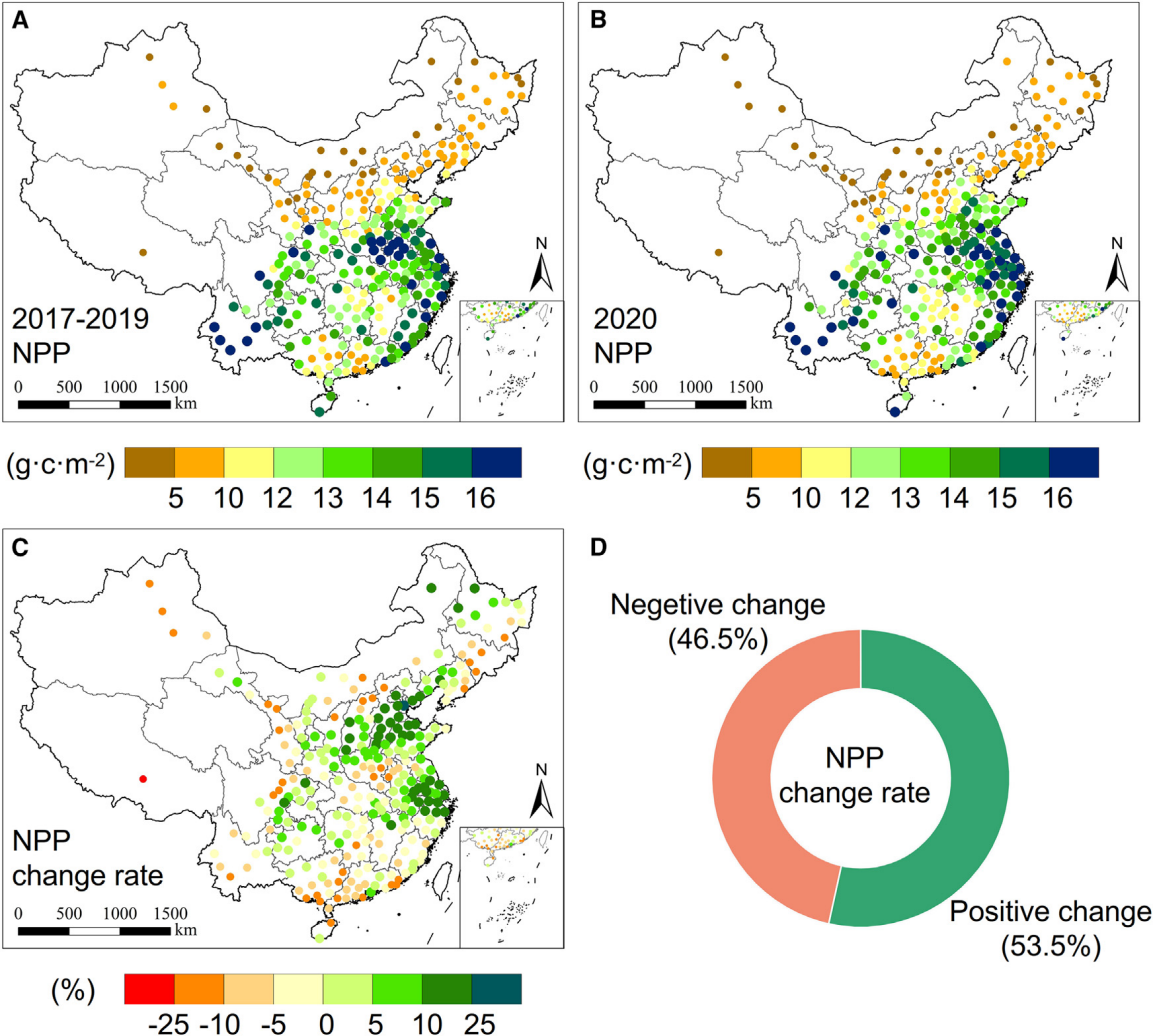
Spatiotemporal distributions of NPP changes during spring of lockdown period in PCC The circular symbols in this figure represent the distribution of locations where 283 PCC are located. (A) NPP distribution in PCC, spring 2017–2019. (B) NPP distribution in PCC, spring 2020. (C) Distribution of NPP change rate in PCC during lockdowns. (D) Proportions of PCC showing positive and negative changes in NPP.

The distributions of spring NPP for the years 2017–2019 and 2020 were obtained through the processing and analysis of remote sensing images (Figures 1A and 1B). This enabled us to investigate the spatiotemporal changes in NPP of PCC before and after the lockdown. Vegetation NPP in China exhibited a consistent pattern in the spring, with higher values in coastal cities in the east and south and lower values in inland cities in the west and north. Figure 1C displays the distribution of NPP change rates during the lockdown, whereas the percentage of cities experiencing positive or negative changes is depicted in Figure 1D. It is clear that the number of cities with positive changes in NPP (147 cities) exceeded those with negative changes, accounting for 53.5% of all cities nationwide. The average NPP change rate for all PCC was +1.06% (Table 1), suggesting that vegetation growth in Chinese cities generally improved during the short-term lockdown period.

**Table 1:** Average change rates of NPP and environmental variables among 283 PCC, as well as the proportion of PCC with positive and negative changes

	Mean rate of change (%)	Percentage of positive changes (%)	Percentage of negative changes (%)
NPP	1.06	53.5	46.5
AOD	−1.50	37.1	62.9
NTL	10.41	78.2	21.8
TEM	−0.98	47.3	52.7
PRE	−9.15	33.1	66.9

Positive changes in NPP were primarily observed in the Beijing-Tianjin-Hebei and Yangtze River Delta regions, with significant increases in North and East China. Tianjin experienced the largest positive change (+25.2%). Conversely, Liaoning Province, Guangxi Province, and Tibet saw negative changes in NPP, with several cities in the northeast, south, and west inland regions exhibiting declines. The largest decrease was found in Lhasa city (−25.8%). Overall, NPP in Chinese cities showed an increasing trend, with considerable positive changes in northern and eastern cities and relatively lower negative changes in southern and western regions.

### Spatiotemporal changes in environmental variables in PCC

**Figure 2:**
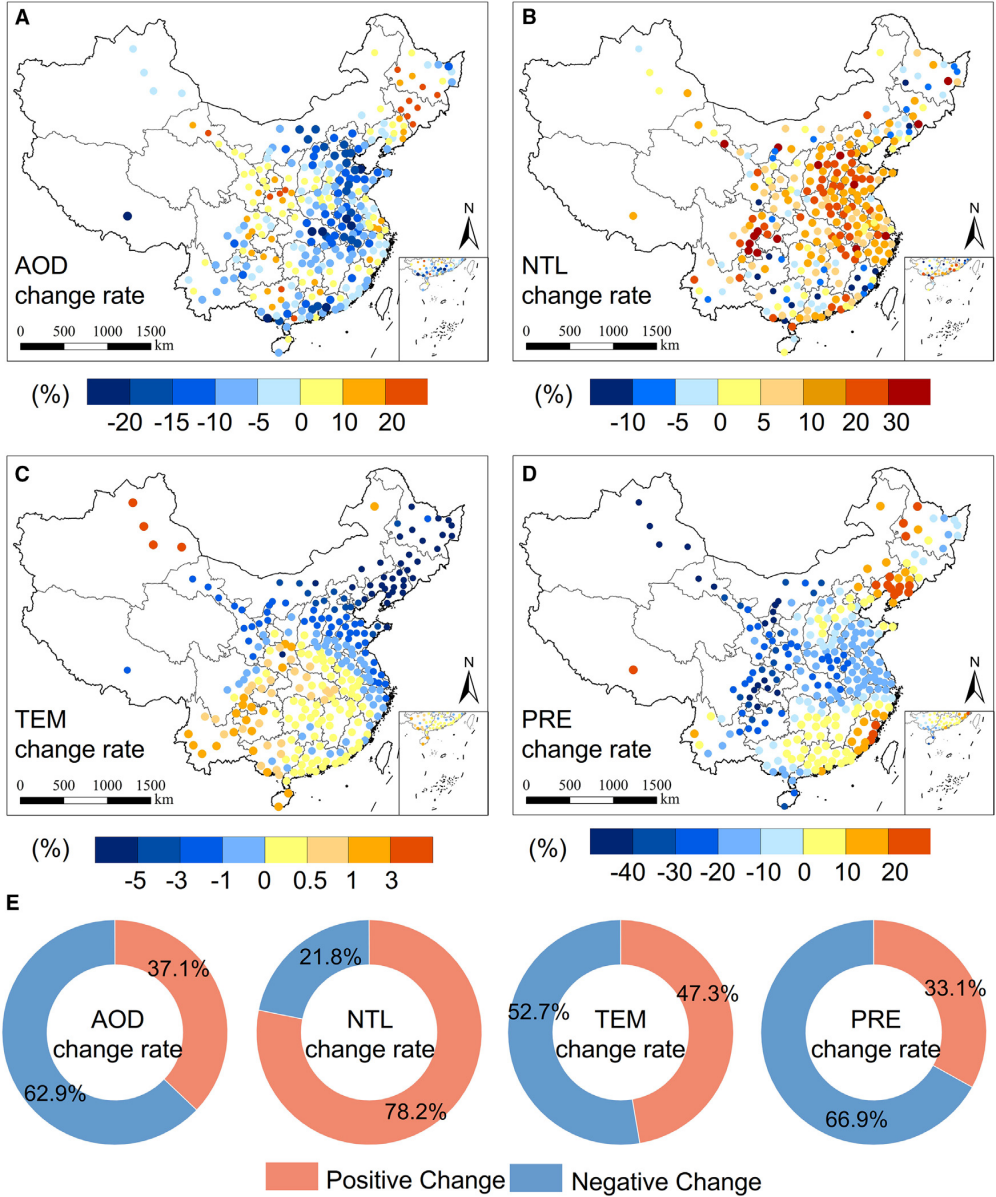
Spatiotemporal distributions of environmental variables changes during spring of lockdown period in PCC The circular symbols in this figure represent the distribution of locations where 283 PCC are located. (A) Distribution of AOD change rate in PCC during lockdowns. (B) Distribution of NTL change rate in PCC during lockdowns. (C) Distribution of TEM change rate in PCC during lockdowns. (D) Distribution of PRE change rate in PCC during lockdowns. (E) Proportions of PCC showing positive and negative changes in environmental variables.

The spatiotemporal variations of several environmental variables that could impact NPP before and after the lockdown were examined in this study. Figure 2 presents the distributions of changes in AOD, NTL, TEM, and PRE, as well as the percentages of cities with positive and negative changes.

In particular, AOD exhibited widespread negative changes, with 62.9% of PCC experiencing decreases, far exceeding those with increases (Figures 2A–2E). The average AOD change rate for all PCC was −1.50% (Table 1), indicating a general decline in AOD and an improvement in air quality nationwide during the lockdown period. Significant declines in AOD were noted in Hebei and Shandong provinces in North China, Hubei Province in Central China, and Anhui Province in Eastern China. Nevertheless, some increases were observed in cities in Heilongjiang and Jilin provinces in Northeast China, Shaanxi and Gansu provinces in Northwest China, and parts of the eastern and southern coastal regions. Xiaogan City had the largest negative change (−28.3%), while Liuzhou City had the biggest positive change (63.5%). Overall, AOD levels in PCC showed a downward trend, with notable declines in Central and Northern areas and marginal increases in Southern and Western regions.

Additionally, as shown in Figures 2B–2E, NTL saw a substantial increase, with 78.2% of cities experiencing positive changes. The average NTL change rate across all PCC was +10.41% (Table 1). Cities in North, Central, and East China, as well as some in Sichuan Province, experienced increases in NTL. Yibin City showed the largest rise (+91.5%), while negative changes were mainly observed in cities in Fujian and Guangxi provinces, with Hechi City experiencing the most significant drop (−22.6%).

Furthermore, TEM changes were more variable, with 52.7% of cities seeing negative changes during the lockdown period (Figures 2C–2E), with an average drop of 0.98% (Table 1). This trend was particularly pronounced in cities in Northeast China, while the Southwest and Xinjiang regions showed increases, indicating an overall trend of increasing TEM from Northeast to Southwest. PRE decreased by 9.15% across the study area (Table 1), with 66.9% of cities experiencing negative changes (Figures 2C–2E). Most cities saw negative changes, except for a few in Northeast China and the southern coastal regions.

### Relationship among industrial level, traffic level, NPP, and environmental variables in PCC

This study examined how traffic volume and industrial development, the two most impacted aspects of urban life during the COVID-19 lockdown, influenced vegetation and environmental variables in PCC. We assigned each PCC two basic urban characteristics (BUC): traffic level (TrL) and industrial level (InL). Table S2 lists these values for parts of the 283 PCC.

**Figure 3:**
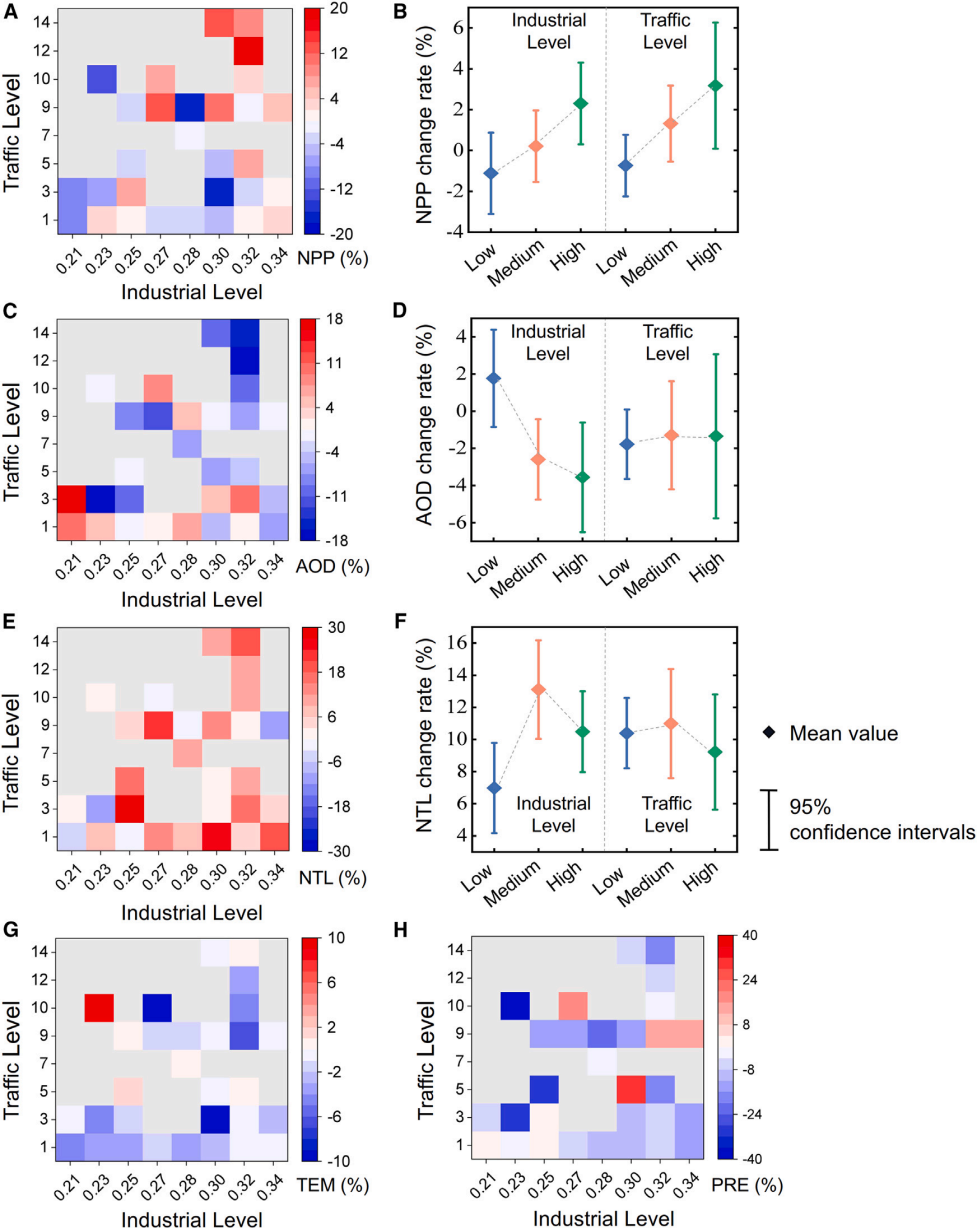
The relationships among parameter change and urban industrial and traffic levels (A) Heatmap of NPP change rate with industrial and traffic levels. (B) Interval plot of NPP change rate across different industrial and traffic grades. (C) Heatmap of AOD change rate with industrial and traffic levels. (D) Interval plot of AOD change rate across different industrial and traffic grades. (E) Heatmap of NTL change rate with industrial and traffic levels. (F) Interval plot of NTL change rate across different industrial and traffic grades. (G and H) Heatmap of TEM change rate and PRE change rate with industrial and traffic levels, respectively. In interval plots, the diamonds represent the mean values and the different colored intervals represent the 95% confidence intervals. The mean (Mean value) was used to represent the central tendency, the standard deviation (SD) to indicate dispersion, and the 95% confidence interval (95% CI) was calculated using a two-tailed t-distribution.

The heatmap makes it straightforward to figure out the relationships between various parameter change rates and BUC. Of these, a rising tendency for NPP change rate with increasing InL and TrL is shown in Figure 3A, while Figure 3C displays a general decline in AOD change rate with increasing InL and TrL. This suggests a negative correlation between AOD and BUC and a positive correlation between NPP change and BUC in PCC during lockdowns. However, the change patterns for NTL, TEM, and PRE were not significant in relation to BUC (Figures 3E–3G and 3H).

We further categorized both InL and TrL into three grades—high, medium, and low—using the Natural Breaks (Jenks) method,^43^ considering PCC’s share of industrial output and public transportation ridership, to explore these relationships in greater depth. Table S3 shows the classification rules, and Table S2 lists the urban grades for the part of the 283 PCC. Interval plots were used to illustrate the NPP, AOD, and NTL change rates of cities with varying traffic and industry grades (Figure 3).

Cities with a High InL had NPP, AOD, and NTL change rates of 2.30% ± 9.39%, −3.56% ± 13.83%, and 10.49% ± 11.80%, respectively. Medium-level cities had NPP, AOD, and NTL change rates of 0.21% ± 9.02%, −2.59% ± 11.13%, and 2.48% ± 15.76%, respectively. Low-level cities showed changes of −1.12% ± 9.19% for NPP, 1.78% ± 12.07% for AOD, and 6.98% ± 12.93% for NTL. Regarding TrL, High-level cities had NPP, AOD, and NTL change rates of 3.18% ± 9.67%, −1.34% ± 13.82%, and 9.22% ± 11.20%, respectively. Medium-level cities showed changes of 1.32% ± 8.64% for NPP, −1.29% ± 13.49% for AOD, and 10.99% ± 15.73% for NTL. Low-level cities had changes of −0.74% ± 9.34% for NPP, −1.78% ± 11.57% for AOD, and 10.40% ± 13.57% for NTL.

Both NPP change rates exhibited significant positive trends with InL and TrL (Figure 3B). AOD change rates showed a significant negative trend with InL but not with TrL (Figure 3D). In particular, we found that during lockdowns, cities with higher InL exhibited more substantial negative changes in AOD and more significant positive changes in NPP. Cities with greater TrL saw notable improvements in NPP. Figure 3F indicates no significant correlation between NTL change rates and the two characteristics, with the medium level showing higher variation rates.

### Drivers of vegetation change in PCC

**Figure 4:**
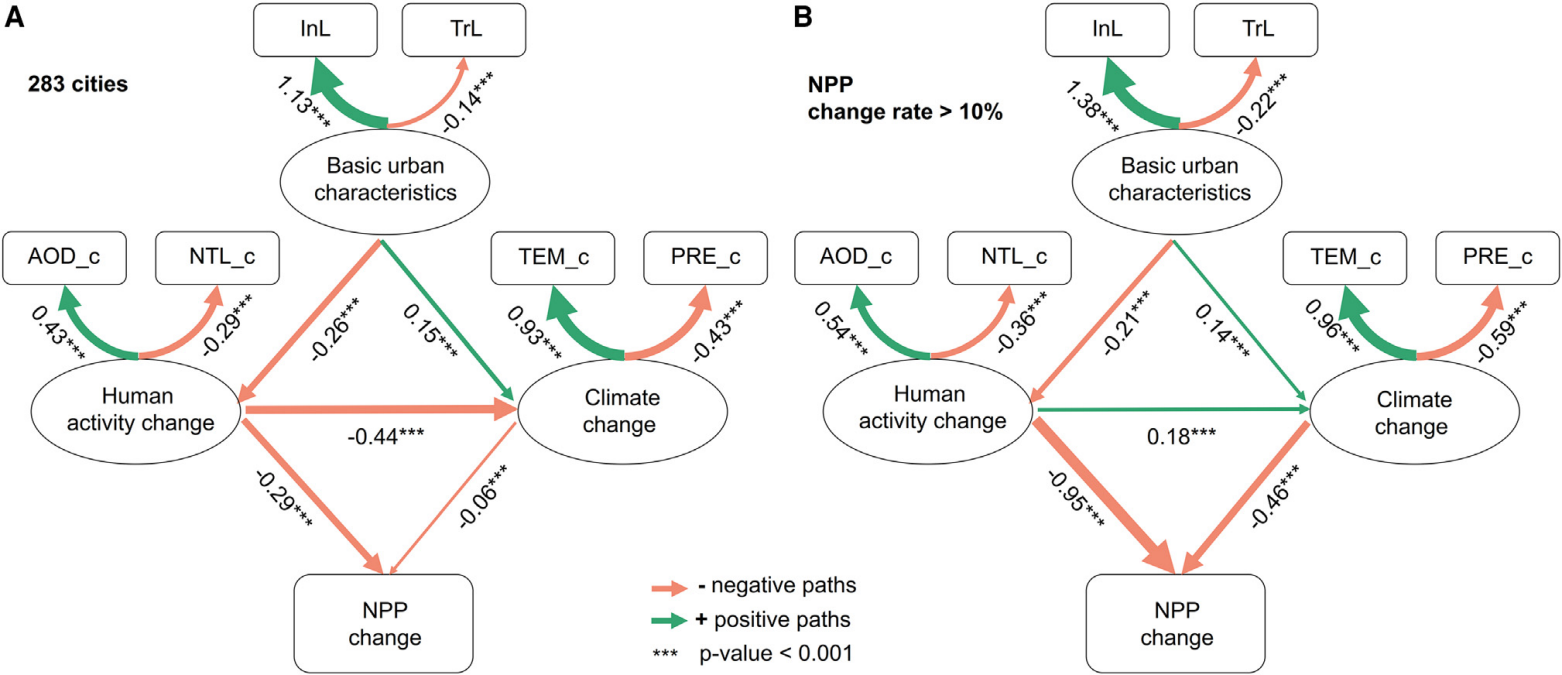
SEM results of NPP changes in PCC during the lockdown period Squares represent observable variables, ellipses represent latent variables. Green lines indicate positive paths, red lines indicate negative paths. (A) SEM result for 283 PCC nationwide. (B) SEM result for PCC with NPP change rates greater than 10%. ∗∗∗ *p* value <0.001 (Wald Z-test based on maximum likelihood estimation).

The results of fitting structural equation modeling (SEM) for 283 PCC nationwide are displayed in Figure 4A. The model’s fit optimization indexes (the comparative fit index (CFI) = 0.95, the root-mean-square error of approximation (RMSEA) = 0.05, the standardized root-mean-square residual (SRMR) = 0.06) all satisfy the significance thresholds. The effects of basic urban characteristics (BUC), human activity change (HAC), climate change (CC) on NPP change are detailed in Table 2. HAC had the most significant impact, with total effects of −0.26 on NPP change. BUC influenced HAC and CC, which had an indirect impact on NPP changes, with essentially no direct effect. While HAC had a direct negative effect on NPP changes (path coefficient = −0.29) and an indirect positive effect by influencing CC. The direct negative effect of CC on NPP change was only −0.06.

**Table 2:** Based on statistically significant SEM paths, the direct, indirect, and total effects of the basic urban characteristics (BUC), human activity change (HAC), and climate change (CC) on NPP change for 283 PCC and cities with NPP change rates greater than 10%

City category		Direct		Indirect		Total	
		Paths	Coefficients	Paths	Coefficients	Paths	Coefficients
283 PCC	Effects on human activity change	BUC→HAC	−0.26			BUC→HAC	−0.26
	Effects on climate change	BUC→CC HAC→CC	0.15 −0.44	BUC→HAC→CC	0.11	BUC→CC HAC→CC	0.26 −0.44
	Effects on NPP change	HAC→NPP CC→NPP	−0.29 −0.06	BUC→HAC→NPP BUC→HAC→CC→NPP BUC→CC→NPP HAC→CC→NPP	0.08 −0.01 −0.01 0.03	BUC→NPP HAC→NPP CC→NPP	0.06 −0.26 −0.06
NPP change rate >10%	Effects on human activity change	BUC→HAC	−0.21			BUC→HAC	−0.21
	Effects on climate change	BUC→CC HAC→CC	0.14 0.18	BUC→HAC→CC	−0.04	BUC→CC HAC→CC	0.1 0.18
	Effects on NPP change	HAC→NPP CC→NPP	−0.95 −0.46	BUC→HAC→NPP BUC→HAC→CC→NPP BUC→CC→NPP HAC→CC→NPP	0.20 0.02 −0.06 −0.08	BUC→NPP HAC→NPP CC→NPP	0.16 −1.03 −0.46

Overall analysis of cities nationwide may not yield significant results due to spatial heterogeneity.^44^ To further explore driving mechanisms within PCC with notable NPP responses during the lockdown (NPP change rate >10%), we fitted an SEM model to these cities (CFI = 0.99, RMSEA = 0.04, SRMR = 0.07), with results shown in Figure 4B. The contribution of PCC with NPP change rate >10% to overall NPP change during COVID-19 is detailed in Table S4. The total effects of BUC, HAC, and CC on NPP changes were 0.16, −1.03, and −0.46, respectively. HAC remained to have the largest impact, and the intensity of this impact was more pronounced compared to all PCC. HAC had a direct effect on NPP changes with a path coefficient of −0.95, as well as an indirect positive effect through CC. CC also produced a large direct negative effect on changes in NPP (path coefficient = −0.46). Furthermore, BUC influenced HAC and CC, indirectly impacting NPP change.

For both the 283 PCC and those with significant NPP changes, InL was the most indicative variable of city characteristics during COVID-19, followed by TrL (coefficients of 1.13 and 1.38, respectively). And AOD change (AOD_c) was a more accurate indicator of changes in human activities than NTL change (NTL_c) (coefficients of 0.43 and 0.54, respectively). Both PRE change (PRE_c) and TEM change (TEM_c) were reliable indicators of CC. According to SEM results, in PCC nationwide and those with major NPP changes, BUC primarily exerted a negative impact on HAC, dominated by AOD_c through InL, thereby negatively influencing NPP change. Therefore, PCC with higher industrial levels saw a larger drop in AOD during the lockdown, leading to more notable positive changes in NPP.

## Discussion

### Differences in changes in vegetation and environmental variables in PCC with varying industrial and traffic levels

This study reveals the overall trends in vegetation and environmental variables in China during the COVID-19 lockdown by analyzing the change rates of various parameters across cities. The improvement in NPP aligns with previous studies,^7^ and our findings suggest that air quality in PCC improved during the lockdown, supported by existing studies.^45^^,^^46^ The increase in NTL indicates a high level of urban activity following the epidemic, reflecting a resumption of labor and output that even surpassed previous years’ levels, which is similar to some studies.^47^ However, previous studies have primarily focused on national scales or individual cities, lacking in-depth research on variations among different cities in China.

Building on prior research, this study examines the relationship between two basic urban characteristics and variations in NPP during the lockdown period. It also illustrates the varying degrees to different city categories were affected by lockdown measures, as well as how the environment and vegetation responded differently to COVID-19 lockdown. Basic urban characteristics include InL and TrL, both of which were greatly impacted in PCC during the lockdown.^2^ We used the gross industrial production to GDP ratio to represent industrial growth,^48^ excluding primary and tertiary industry values due to their minimal relevance to lockdown impacts. Additionally, the connection between air pollution and city traffic volume during the lockdown has been confirmed.^46^ After taking into account the large amounts of missing data on private vehicle ownership found in local statistics yearbooks, we finally selected publicly accessible data on the passenger traffic volume on public vehicles to represent city traffic levels.

During the COVID-19 lockdown, factory production and transportation in mainland China faced stringent restrictions of comparable severity.^49^ These constraints on economic activities and mobility directly impacted energy consumption patterns, leading to a notable reduction in environmental pollution.^50^ Prior studies have identified industrial and transportation emissions as the primary contributors to AOD formation,^22^ with cities exhibiting higher industrial and traffic intensities typically experiencing more severe air pollution.^45^ Consequently, AOD demonstrated a widespread declining trend across Chinese cities, highlighting the negative correlation between urban industrial and traffic scales and AOD fluctuations (Figure 3). The concentration of heavy industries and coal-fired power plants in central and northern China partly explains the sharp decrease in AOD observed during the lockdown in provinces, such as Hebei, Shandong, and Hubei.^51^ In contrast, southern regions, dominated by light industries and service-oriented economies, exhibited more modest changes in AOD levels. The anomalous rise in AOD in northeastern cities was likely driven by seasonal sandstorms and centralized heating systems.^28^^,^^52^ The increase in NTL intensity was unexpected but can be attributed to heightened residential energy consumption and the partial resumption of industrial operations after initial shutdowns. Industrial recovery was prioritized in northern and eastern cities, resulting in a more pronounced rise in nighttime light intensity and signaling the restoration of economic activity.^47^

The reduction in AOD decreased atmospheric particulate matter concentrations, allowing greater solar radiation to reach vegetation. This improvement in light conditions enhanced photosynthesis and stimulated plant growth. Additionally, the diffuse radiation fertilization effect further amplified this benefit, as reduced aerosols increased the proportion of diffuse sunlight, which is more efficient for photosynthesis.^32^^,^^35^^,^^41^ As a result, NPP exhibited a significant upward trend during and after the lockdown, supporting the observed positive correlation between urban NPP changes and industrial and traffic levels (Figure 3). In industrial hubs such as the Beijing-Tianjin-Hebei region and the Yangtze River Delta, strict lockdown measures triggered substantial emission reductions, markedly improving air quality. Combined with favorable spring climatic conditions, these factors led to more substantial NPP increases in these areas. Tianjin, characterized by its heavy industrial base and dense transportation network, was particularly impacted by the lockdown, resulting in the most significant NPP growth.^53^ However, it is noteworthy that NPP in Northeast and South China did not follow this increasing trend. In Northeast China, lower TEM may have slowed plant metabolic rates, potentially counteracting the positive effects of improved air quality. Meanwhile, in southern regions, the reduction in PRE likely limited water availability, constraining vegetation growth (Figure 4). This phenomenon may also be associated with abnormally high AOD levels and the extensive coverage of evergreen forests in these regions.^6^

Additionally, to better understand the underlying drivers of NPP changes, previous studies have primarily focused on climatic factors, with limited consideration of human influences. Traditional approaches, such as linear regression, partial correlation analysis, residual analysis, and geographical detectors, have been widely used to assess the impacts of natural and anthropogenic factors on vegetation dynamics.^31^^,^^54^^,^^55^ However, these methods often fall short in capturing the complex and interconnected relationships among multiple variables. SEM offers a more robust framework for revealing hidden relationships behind vegetation changes, as it can quantitatively assess both direct and indirect effects of influencing factors.^56^ This makes SEM particularly well-suited for analyzing intricate causal networks in ecosystems.^57^ SEM results from this study indicate that HAC exerts the most significant direct and total influence on NPP variations, primarily through the reduction of AOD. This finding suggests that restrictions on industrial and transportation activities during the lockdown indirectly boosted vegetation productivity by improving air quality.^41^^,^^58^ In cities that experienced substantial increases in NPP, a closer examination of their urban structural characteristics reveals a higher proportion of industrial and traffic activities. This composition amplified the indirect positive effects on NPP, as these cities saw more significant reductions in AOD due to stricter lockdown measures. Furthermore, the indirect positive impact of HAC on CC suggests that decreased human activities also influenced local climate conditions, leading to moderated temperature and precipitation patterns that further supported vegetation growth. This insight aligns with previous research on the indirect climate effects of reduced human activities.^59^ Interestingly, in cities with notable NPP increases, the combined effects of HAC and CC were more pronounced, indicating that the synergistic interaction between reduced human disturbances and favorable climatic conditions jointly enhanced vegetation productivity.^41^

The results of this study can provide valuable insights for future urban planning and policy decisions. It emphasizes the importance of regulating industrial and transportation activities to improve air quality and enhance vegetation productivity. Policies aimed at reducing emissions during industrial and traffic peak periods help alleviate pollution and support sustainable urban development, especially in cities with high industrial density. Furthermore, integrating green spaces into urban development and combining air quality management with the development of green infrastructure can sustainably increase urban vegetation productivity in the long term. These insights can guide effective environmental management strategies in post-pandemic urban recovery efforts.

### Analysis of vegetation change heterogeneity and drivers among different urban agglomerations

Due to the vast number of cities in China, spatial differences in climate, environment, development levels, and industrial structures create distinct patterns in NPP changes during the COVID-19 lockdown. Understanding these regional variations is essential for identifying the key drivers behind NPP dynamics.

Urban agglomerations—economically connected city clusters—are critical in urban studies due to their diverse industrial structures and geographic conditions, which influence human activity and climate impacts on vegetation.^60^^,^^61^ This study examines six major agglomerations: Beijing-Tianjin-Hebei (BTH), Yangtze River Delta (YRD), Pearl River Delta (PRD), Yangtze River Middle-Reach (YRMR), Cheng-Yu (CY), and Middle and South Liaoning (MSL), which are strategically significant for national development.^62^ Details of cities included in these agglomerations are listed in Table S5.

**Figure 5:**
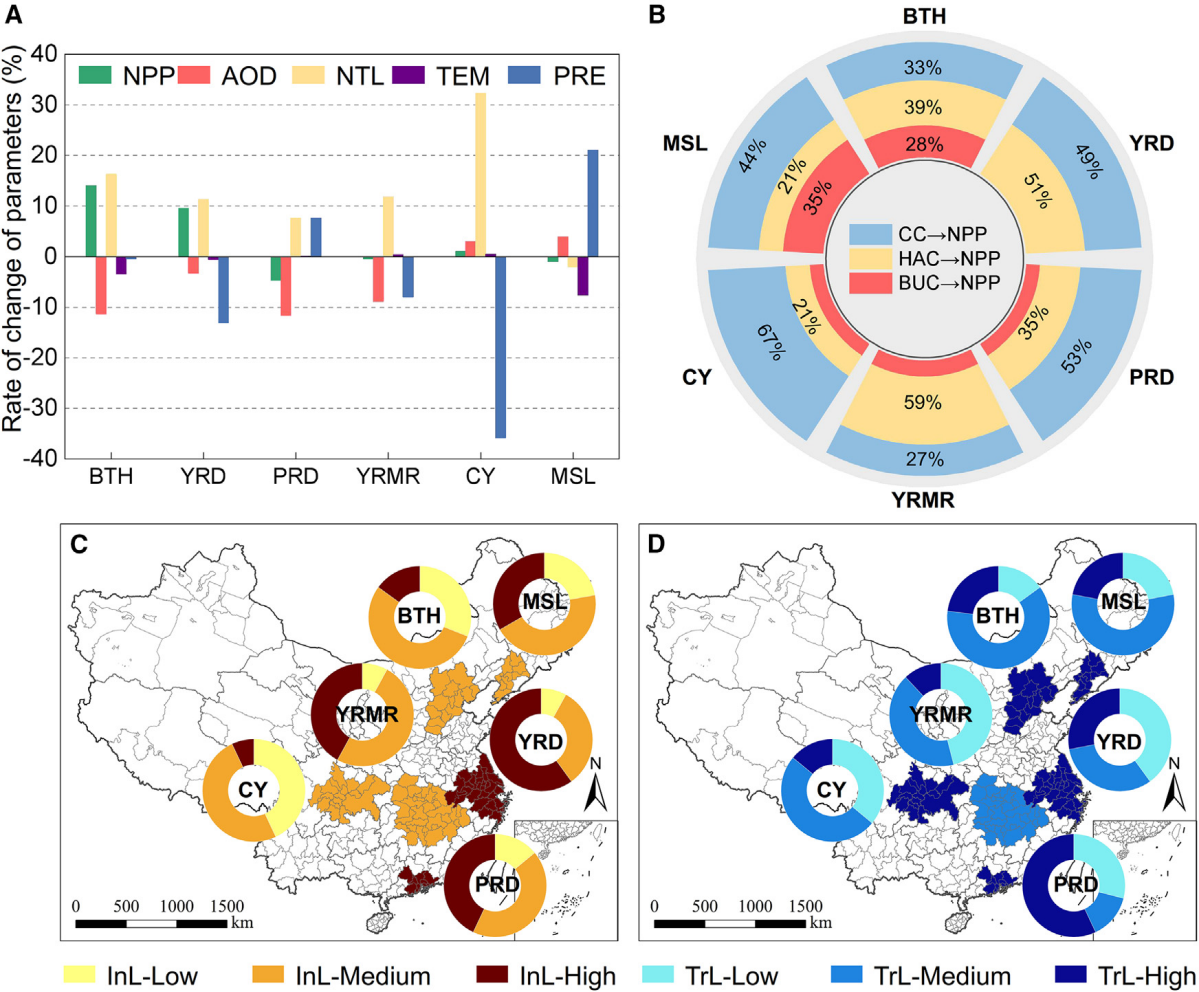
Results of the six urban agglomerations (A) Mean values of the rate of change of each parameter in the six urban agglomerations during lockdowns. (B) Percentages of total effects of three latent variables on NPP in the six urban agglomerations. (C) Average industrial grade and proportions of cities at different industrial grades in the six urban agglomerations. (D) Average traffic grade and proportions of cities at different traffic grades in the six urban agglomerations.

NPP changes across agglomerations: BTH (+14.06%), YRD (+9.58%), and CY (+1.02%) showed NPP increases, while PRD (−4.69%), YRMR (−0.45%), and MSL (−0.94%) experienced declines (Figure 5A). SEM analysis (Table S6) reveals the direct, indirect, and total effects of influencing factors on NPP changes. And the percentages of total effects of three latent variables on NPP changes within each urban agglomeration are presented in Figure 5B.

As a heavily industrialized region,^63^ BTH saw a sharp AOD drop (−11.28%) linked to reduced industrial and traffic activities, aligning with previous findings. SEM results show that HAC had the greatest impact on NPP (39%), with medium-to-high industrial and traffic levels amplifying this effect (Figures 5B–5D). Notably, Qinhuangdao’s coal throughput hit a four-year low during the lockdown, highlighting how reduced industrial activity significantly lowered AOD and boosted NPP.^50^ Anthropogenic aerosols are predominant in the BTH area, which is linked to the notable decline in AOD.^28^

YRD, with severe air pollution,^64^ saw a notable AOD reduction (−3.26%) during the lockdown.^28^ TEM decreased slightly, while PRE showed a larger negative change (−13.10%) (Figure 5A). SEM analysis indicates that HAC and CC contributed 51% and 49% to NPP growth, respectively. Despite its high industrial-traffic grade, BUC had minimal influence, possibly due to the dominance of less-affected technology and capital-intensive industries.^65^

CY, the most economically developed region in southwestern China,^62^ recorded a slight NPP increase despite an AOD rise (+2.98%) and a substantial PRE decline (−35.83%) (Figure 5A). SEM results highlight CC (67%) as the dominant driver of NPP change, with minor effects from HAC and BUC. CY’s sparse population, weaker industry,^18^ and hilly terrain hinder pollutant dispersion, contributing to the AOD increase.^66^

With a pleasant climate and a high concentration of industries,^67^ PRD’s high industrial-traffic grade saw a moderate AOD decline (−3.26%). However, SEM analysis reveals CC (53%) as the primary driver of NPP reduction. Limited industrial restrictions and light industry dominance likely reduced BUC’s impact on NPP, while PRE increased (+7.65%), slightly mitigating the decline (Figure 5A).^68^

MSL, in Northeast China, experienced an AOD rise (+3.94%), with significant TEM (−7.58%) and PRE (+21.09%) changes.^69^ SEM results show CC (44%) and BUC (35%) as key drivers of NPP decline. Increased precipitation may have hindered pollutant dispersion, while dust storms and centralized heating contributed to AOD growth.^28^^,^^52^

YRMR, including Wuhan, saw an AOD drop (−8.83%) but negligible NPP improvement.^6^ SEM results reveal HAC (59%) as the dominant factor, yet low transportation activity and reduced precipitation limited NPP recovery. The indirect effect of HAC on CC may have outweighed its direct effect on NPP (Table S6).

In conclusion, NPP changes during the lockdown varied across urban agglomerations due to different driving factors. Lockdown measures had a stronger impact on regions with higher traffic and industrial activity, leading to significant reductions in human activities and AOD. Regional drivers of NPP change differed: BTH was mainly influenced by human activity and urban characteristics; YRD and YRMR by both human activity and climate; CY and PRD primarily by climate; and MSL by the combined effects of climate and urban structure. To mitigate environmental impacts, regions should optimize industrial structures and regulate human activities based on local climate and development levels, focusing on reducing industrial and transportation emissions. Promoting industrial transformation and technology-intensive industries can support environmental protection and sustainable development. Understanding these dynamics under atypical human disturbances offers valuable guidance for targeted environmental policies.

### Conclusions

Rapid urbanization and industrialization in China have heavily polluted the environment, negatively affecting vegetation health. The COVID-19 lockdown, as an atypical anthropogenic disturbance, temporarily restricted human activities, improving air quality while disrupting industries and transportation. This study examines the impact of urban industrial and traffic levels on vegetation changes during the lockdown, revealing key interactions among urban features, vegetation, and human activity. The findings offer insights for sustainable environmental protection and enhancing urban vegetation productivity. Key conclusions are as follows:

(1) Over half (53.5%) of PCC experienced increased NPP during the lockdown, with an average rise of 1.06%. Positive changes were notable in northern and eastern China, particularly in the Beijing-Tianjin-Hebei and Yangtze River Delta regions.
(2) Air quality improved as 62.9% of PCC saw an average AOD reduction of 1.50%, especially in Central China. Conversely, 78.2% of PCC showed a 10.41% average increase in NTL, with positive changes concentrated in North, Central, and East China. TEM remained relatively stable, while PRE declined in many cities.
(3) PCC with higher InL and TrL showed steeper NPP improvements and more significant AOD reductions. High-InL cities exhibited notable AOD decreases and NPP increases, while high-TrL cities also saw marked NPP growth.
(4) BUC negatively impacted HAC, primarily through AOD reductions, which in turn negatively affected NPP change. HAC’s total effect on NPP change (−0.26) outweighed that of climatic conditions (−0.06), with a stronger impact observed in cities with higher NPP change rates.

These findings emphasize the need for urban policies that reduce emissions, expand green spaces, and adopt cleaner energy to protect vegetation health. Future research should explore the impact of similar disruptions on urban ecosystems, while integrating lessons from the COVID-19 lockdown into city planning to balance economic growth with ecological sustainability.

### Limitations of the study

This study undertook a comprehensive analysis of vegetation NPP changes in PCC before and after lockdown measures, focusing on industrial and traffic levels. However, our definition of urban InL was limited to the ratio of gross industrial production output to GDP, without considering other crucial industrial attributes like the number of large-scale enterprises, primary industrial types, and industrial emissions. Similarly, our assessment of traffic levels only accounted for passenger volumes on public vehicles, neglecting potential impacts from emissions related to private vehicles. Moreover, different urban industrial zones may experience varying degrees of lockdown effects, potentially influencing vegetation responses in diverse ways, a nuance not fully explored in our study. Therefore, to comprehensively evaluate industrial and traffic dynamics across PCC, future research will leverage more extensive urban statistical data to refine classifications and derive deeper insights.

The data used in this study include multi-source data such as statistical data and remote sensing imagery. During the data processing, there may be information omissions and errors, such as in the process of unifying resolutions. To avoid this issue, future research can utilize other data sources and explore more suitable data processing methods. In addition, our SEM also has limitations. We only focused on observable variables related to transportation and industrial characteristics during the lockdown, neglecting other urban features that may influence vegetation dynamics. Moreover, our model primarily considered urban factors, changes in human activities, and climate, without incorporating other variables that could affect NPP changes, such as topography, soil moisture, CO_2_, and vegetation types. Furthermore, the SEM was constructed based on prior knowledge and previous studies, which may present certain limitations in simulating complex causal relationships. Therefore, to strengthen the SEM and gain a better understanding of the driving factors behind urban vegetation changes during the lockdown, future research needs to include a broader range of relevant factors and optimize the causal relationships within the model.

## Resource availability

### Lead contact

Further information and requests for resources should be directed to and will be fulfilled by the lead contact, Jia Wang (wangjia2009@bjfu.edu.cn).

### Materials availability

This study did not generate new unique materials.

### Data and code availability

• The data used in this study are all available from public resources that have been appropriately cited within the manuscript.
• All custom code can be available on request from the lead contact.
• Any additional information required to reanalyze the data reported in this paper is available from the lead contact upon request.

## Acknowledgments

This work was supported by the 10.13039/100017338Key Program of National Natural Science Foundation of China (No. 42330507), and thank the editors and anonymous reviewers for their kindly view and constructive suggestions.

## Author contributions

Y.L.: data curation, formal analysis, methodology, investigation, project administration, visualization, and writing—original draft. S.H.: conceptualization, data curation, methodology, project administration, writing—review and editing. P.F.: conceptualization, data curation, and methodology. Y. L.: conceptualization, data curation, and methodology. J.W.: conceptualization, funding acquisition, investigation, resources, supervision, and validation.

## Declaration of interests

The authors declare no competing interests.

## STAR★Methods

### Key resources table

**Table:** 

REAGENT or RESOURCE	SOURCE	IDENTIFIER
**Deposited data**		

500m PSNnet	Google Earth Engine	https://lpdaac.usgs.gov/products/mod17a2hv061/
1km AOD	Google Earth Engine	https://lpdaac.usgs.gov/products/mcd19a2v061/
0.004° NTL	Environmental Sciences Chinese Academy of Sciences	http://www.resdc.cn/
1km TEM	Qinghai-Tibet Plateau/Third Pole Environment Data Center	http://data.tpdc.ac.cn/
1km PRE	Qinghai-Tibet Plateau/Third Pole Environment Data Center	http://data.tpdc.ac.cn/
Boundary of the study area	Peng Cheng Laboratory	https://data-starcloud.pcl.ac.cn/zh/resource/14
Industrial output, GDP	Local Statistical Yearbooks	https://tjj.beijing.gov.cn/ et al.
Passenger traffic on public vehicles	Local Statistical Yearbooks	https://tjj.beijing.gov.cn/ et al.

**Software and algorithms**		

Arcgis 10.4	ESRI	https://desktop.arcgis.com/zh-cn/desktop/index.html
R Project	Open-source software	https://www.r-project.org/

### Method details

#### Study area

In this study, 283 PCC with complete data are taken as the study object. It excludes five cities with missing gross industrial production output data (Baishan, Songyuan, Baicheng, Suizhou and Sansha) and seven cities with missing passenger traffic on public vehicles data (Dongguan, Zhongshan, Danzhou, Neijiang, Meishan, Rikaze and Naqu). 283 PCC vary greatly in climate and economy. Eastern cities are highly developed with humid climates, driven by industry and trade. Western cities are arid or mountainous, focusing on energy and agriculture. Southern cities have warm, rainy climates, supporting manufacturing and commerce. Northern cities face cold, dry winters and hot summers, with economies based on agriculture and heavy industry.

Furthermore, the urban areas in this study were taken from the multitemporal global urban boundary (GUB) dataset because lockdowns were largely concentrated within metropolitan boundaries (Figure S1). Approximately 189,292 km^2^, or 1.97% of the nation’s total land area, is covered by the study area. Delineated urban boundaries in GUB data are capable of capturing the geometries of urban extent surrounding urban fringe areas, according to comparisons with other products concerning global urban areas.^70^

#### Data description and processing

NPP serves as a critical component of the terrestrial carbon cycle, directly reflecting the vegetative production capacity under natural environmental conditions.^71^^,^^72^ The NPP data were calculated from the net photosynthesis (PSNnet) data. The PSNnet data were obtained from the Moderate Resolution Imaging Spectroradiometer (MODIS) product MOD17A2, which provides global vegetation monitoring at a 500-meter resolution updated every 8 days.^73^ The algorithm of this dataset has been previously employed for accurate assessment of vegetation productivity in China.^74^^,^^75^

NPP refers to the biomass produced by plants that is available to the rest of the food chain and is calculated as GPP minus autotrophic respiration (Ra).^76^ Based on the MOD17A2 algorithm, plant Ra can be divided into maintenance respiration (Rm) and growth respiration (Rg). The PSNnet value is the difference of the GPP and Rm.^77^ Rg represents the energy cost of constructing organic compounds fixed by photosynthesis and is empirically parameterized as 25% of NPP.^78^ Therefore, NPP can be directly calculated from PSNnet using the following algorithm:

(Equation 1) $$NPP=GPP-R_{a}$$

(Equation 2) $$R_{a}=R_{m}+R_{g}$$

(Equation 3) $${\text{PSN}}_{\text{net}}=\text{GPP}-R_{m}$$

(Equation 4) $$\text{NPP}={\text{PSN}}_{\text{net}}-R_{g}$$

(Equation 5) $$R_{g}=0.25*\text{NPP}$$

(Equation 6) $$\text{NPP}=0.8*{\text{PSN}}_{\text{net}}$$

##### Human activity variables

Aerosol data came from MCD19A2 V6 gridded L2 AOD at 550 nm, produced daily at a 1 km resolution.^79^ This product is a combined MODIS/Terra and MODIS/Aqua product retrieved with the latest version of the multiangle implementation of atmospheric correction (MAIAC) algorithm.^80^ MCD19A2 has been widely utilized in studies of AOD in China and has been shown to closely match ground-based measurement results.^81^

The NTL data sourced from the National Oceanic and Atmospheric Administration (NOAA) were scanned and imaged by the Suomi National Polar Partnership satellite equipped with the Visible Infrared Imaging Radiometer Suite (NPP-VIIRS).^30^ We downloaded the data from the Environmental Sciences Chinese Academy of Sciences (RESDC), which provides NPP-VIIRS satellite nighttime light remote sensing image data from 2012 to the present. The data were processed to generate monthly national nighttime light brightness data at a spatial resolution of 0.004 degrees (http://www.resdc.cn/).

##### Climate variables

The TEM and PRE data used in this study are sourced from the Qinghai-Tibet Plateau/Third Pole Environment Data Center (https://data.tpdc.ac.cn/), providing a Chinese 1-km monthly average temperature and precipitation dataset. Research has evaluated this dataset based on 496 national meteorological stations in China, demonstrating its reliability for climate change studies in China.^82^

Processing involved re-projection, resampling, clipping, outlier removal, and averaging techniques to ensure data accuracy and consistency across variables and sources. Google Earth Engine (GEE) is a powerful cloud computing platform widely used in environmental monitoring, disaster response, and resource management.^83^ In this study, GEE was used to process and evaluate the remote sensing data. Because the study area is large and the majority of the data have a resolution of 1 km, we unified the spatial resolution to 1 km. Additionally, we standardized the temporal resolution to one month due to the possibility that daily variations in parameters during the lockdown period would be minor and trends might not be noteworthy, as well as the substantial amount of data that was needed.

##### Urban statistical data

The Gross Industrial Production Output to GDP ratio for cities defined the urban industrial level.^48^ The transportation level was defined by the passenger traffic volume on public vehicles, expressed in billions of people.^46^ Data were averaged from 2017 to 2019 and sourced from Local Statistical Yearbooks. Specific datasets used are detailed in Table S1.

#### Structural equation modeling

Structural equation modeling (SEM), which is a robust multivariate technique used in scientific research to analyze causal relationships among variables, has increasingly been applied in ecological studies.^84^^,^^85^ It integrates techniques such as factor analysis, regression, path analysis, and covariance modeling,^86^ which allows for managing both observable and latent variables that cannot be directly measured.^42^ This approach enhances our understanding of complex causal relationships affecting vegetation changes and quantifies direct and indirect effects among variables.^41^^,^^56^ Evaluation of SEM relies on fitting indices to test path coefficients and overall model fit.^84^ When the standardized root mean square residual (SRMR) is close to 0.08, the root mean square error of approximation (RMSEA) is close to 0.06, and the comparative fit index (CFI) approaches 0.95, it indicates a good model fit.^87^ Results of SEM are typically depicted through path diagrams.

Based on a literature review of NPP drivers, we hypothesize that changes in human activities and climate during the pandemic influence NPP. Urban characteristics may indirectly affect NPP by shaping human behavior and local climate conditions. In our model, latent variables that are not observable directly include basic urban characteristics (BUC), human activity change (HAC), and climate change (CC). The observable variables that indicate BUC include industrial level (InL) and traffic level (TrL). HAC is represented by AOD change (AOD_c) and NTL change (NTL_c), and CC is represented by TEM change (TEM_c) and PRE change (PRE_c). These overarching hypotheses were converted into a graphical conceptual model (Figure S2) that illustrates the interactive relationships between driving factors and NPP changes in PCC during the COVID-19 period.

Both direct and indirect impacts are included in the overall impact of latent factors on NPP change.^88^ The direct impact of a latent variable on NPP change is represented by the path coefficient. A larger path coefficient indicates a stronger influence of that variable on NPP change. Indirect effects occur when the path coefficient from a latent variable to an intermediate variable is multiplied by the path coefficient from the intermediate variable to NPP change.^41^

### Quantification and statistical analysis

In this study, we used R, ArcGIS 10.4, and Origin 2021 for data analysis.

ArcGIS 10.4 was used for sampling and coordinate transformation of all remote sensing data to standardize the data resolution and coordinate system. The "Zonal Statistics as Table" tool in ArcGIS 10.4 was applied to calculate the parameter values at the prefecture-level city scale.

Origin 2021 was used for data analysis and result visualization. Descriptive statistical parameters: the mean (Mean value) was used to represent the central tendency, the standard deviation (SD) to indicate dispersion, and the 95% confidence interval (95% CI) was calculated using a two-tailed t-distribution. The experimental results and statistical details can be found in Figure 3, the figure legend, and the results section.

R was used to construct the SEM model to explore the direct and indirect effects of variables on NPP changes. Statistical tests: ∗∗∗ *p*-value < 0.001 (Wald Z-test based on maximum likelihood estimation). The value of n represents the number of prefecture-level cities, *n* = 283/43. The experimental results and statistical details can be found in Figure 4, the figure legend, and the results section.
