# Supplementary material for: Human activity’s impact on urban vegetation in China during the COVID-19 lockdown: An atypical anthropogenic disturbance
Source: iScience. 2025 Mar 11;28(4):112195. doi: 10.1016/j.isci.2025.112195 (PMC11987675; doi:10.1016/j.isci.2025.112195)
Supplement: Document S1. Figures S1 and S2 and Tables S1–S6 [file mmc1.pdf]

iScience, Volume *28*

Supplemental information

Human activity's impact on urban vegetation in China during the COVID-19 lockdown: An atypical anthropogenic disturbance

Yujie Li, Shaodong Huang, Panfei Fang, Yuying Liang, and Jia Wang

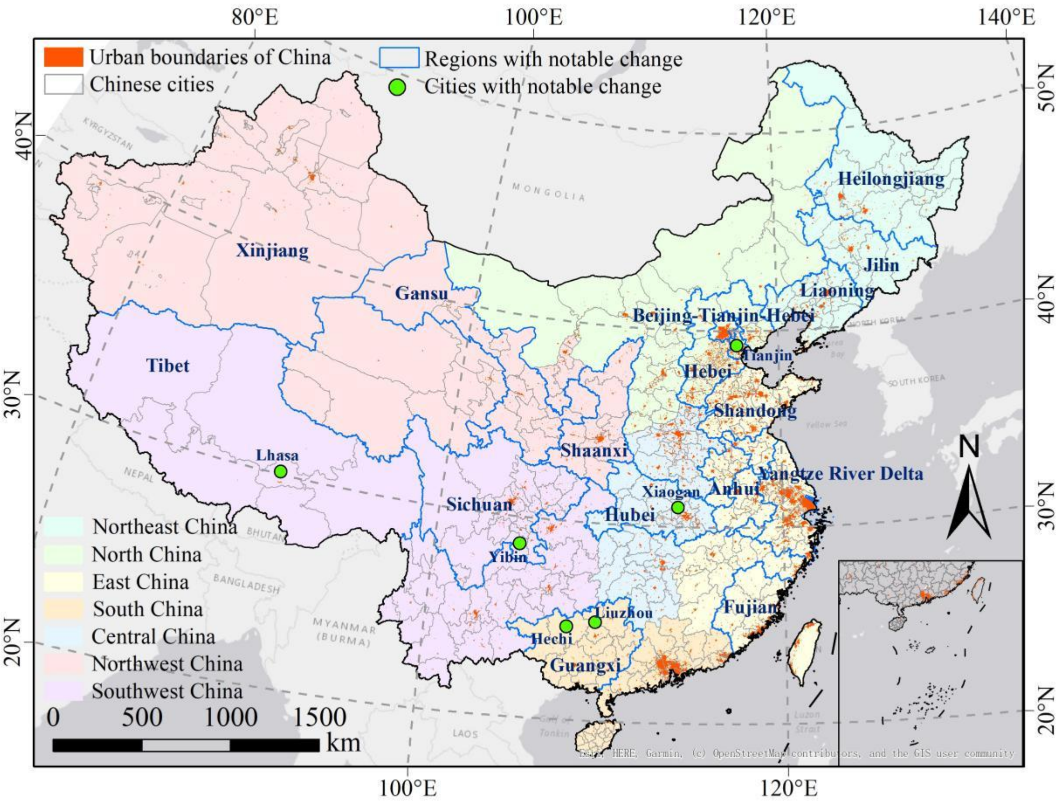

Figure S1. Geographical Location and City Distribution of the Study Area. Although this study focuses on the dynamics of NPP and environmental variables across all prefecture-level cities in China, certain cities and regions (e.g., Tianjin, Hebei Province, and the Yangtze River Delta) exhibited significant changes during the COVID-19 lockdown, which will be further discussed in the Results section. These cities and regions are highlighted in the map.

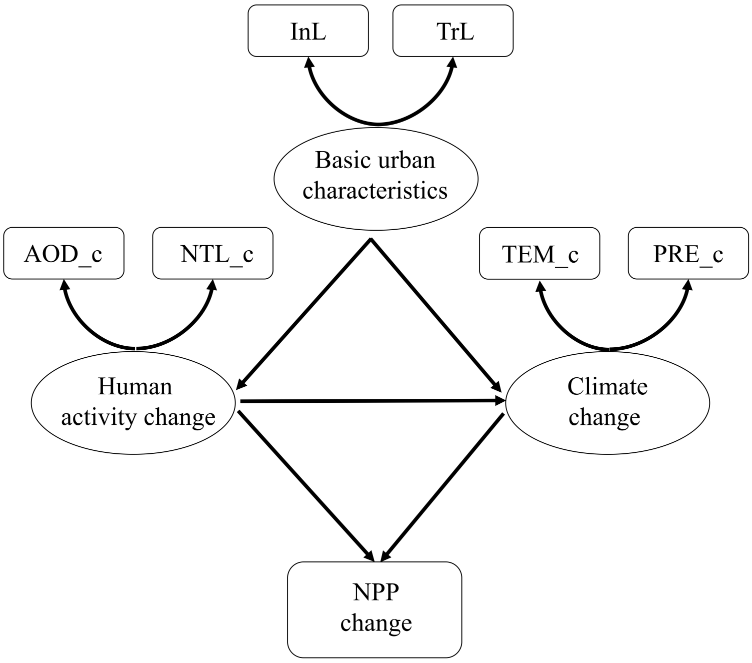

Figure S2. Conceptual structural equation model illustrating NPP changes in PCC during the lockdown period. Squares denote observable variables, while ellipses represent latent variables. Arrows connecting latent variables, as well as those linking latent variables to NPP change, indicate causal relationships, while arrows from latent variables to observable variables represented the correlation relationships between them. The abbreviations of the variables are as follows: InL, industrial level, TrL, traffic level, AOD_c, AOD change, NTL_c, NTL change, TEM_c, TEM change, and PRE_c, PRE change.

Table S1

Datasets catalog introduction.

Variables	Data	Spatial and Temporal Resolution	Data Availability
PSNnet	MOD17A2H	500m/8 days	https://lpdaac.usgs.gov/products/mod17a2hv061/
AOD	MCD19A2	1000m/1 day	https://lpdaac.usgs.gov/products/mcd19a2v061/
NTL	NPP-VIIRS	0.004° /1 month	http://www.resdc.cn/
TEM	Mean temperature dataset	1000m/1 month	http://data.tpdc.ac.cn/
PRE	Mean precipitation dataset	1000m/1 month	http://data.tpdc.ac.cn/
Industrial Level	Industrial output, GDP	-	Local Statistical Yearbooks
Traffic Level	Passenger traffic on public vehicles	-	Local Statistical Yearbooks

Table S2

Levels and grades of industry and traffic in 283 cities across the country.

City	Industrial Level	Industrial Grade	Traffic Level (hundred million people)	Traffic Grade
Beijing	0.139	Low	32.265	High
Tianjin	0.316	Medium	11.964	High
Shijiazhuang	0.407	High	4.036	High
Tangshan	0.475	High	2.075	Medium
Qinhuangdao	0.274	Low	1.108	Medium
Handan	0.372	Medium	1.502	Medium
Xingtai	0.369	Medium	0.931	Medium
Baoding	0.346	Medium	1.288	Medium
Zhangjiakou	0.253	Low	1.404	Medium
Chengde	0.296	Low	0.987	Medium

Table S3

Rules for classifying industry and traffic levels in cities.

Industrial Level		Traffic Level	
Range	Grade	Range (hundred million people)	Grade
>0.4	High	>3	High
0.3~0.4	Medium	0.9~3	Medium
<0.3	Low	<0.9	Low

Table S4

Contribution of cities with NPP change rate > 10% to overall NPP change during COVID-19.

	Number of cities	Amount of NPP change ($g\cdot c\cdot m^{-2}$)
NPP change rate > 10%	43	69.55
NPP Positive Change	147	121.56
Proportion	29.25%	57.21%

Table S5

Cities included in the six urban agglomerations.

Urban agglomerations	Included cities
BTH	Beijing, Tianjin, Shijiazhuang, Tangshan, Qinhuangdao, Handan, Xingtai, Baoding, Zhangjiakou, Chengde, Cangzhou, Langfang, Hengshui
YRD	Hefei, Wuhu, Maanshan, Tongling, Anqing, Chuzhou, Chizhou, Xuancheng, Nanjing, Wuxi, Changzhou, Suzhou, Nantong, Yancheng, Yangzhou, Zhenjiang, Taizhou, Shanghai, Hangzhou, Ningbo, Jiaxing, Huzhou, Shaoxing, Jinhua, Taizhou, Zhoushan
PRD	Guangzhou, Shenzhen, Zhuhai, Foshan, Jiangmen, Zhaoqing, Huizhou
YRMR	Wuhan, Huangshi, Yichang, Xiangyang, Ezhou, Jingmen, Xiaogan, Jingzhou, Huanggang, Xianning, Changsha, Zhuzhou, Xiangtan, Hengyang, Yueyang, Changde, Yiyang, Loudi, Nanchang, Jingdezhen, Pingxiang, Jiujiang, Xinyu, Yingtan, Ji'an, Shangrao, Yichun, Fuzhou
CY	Chongqing, Chengdu, Zigong, Luzhou, Deyang, Mianyang, Suining, Leshan, Nanchong, Yibin, Guangan, Dazhou, Yaan, Ziyang
MSL	Shenyang, Dalian, Anshan, Fushun, Benxi, Yingkou, Liaoyang, Panjin, Tieling

Table S6

Based on statistically significant SEM paths, the direct, indirect, and total effects of the basic urban characteristics (BUC), human activity change (HAC), and climate change (CC) on NPP change for six urban agglomerations.

Urban agglomerations		**Direct**		**Indirect**		**Total**	
		Paths	Coefficients	Paths	Coefficients	Paths	Coefficients
BTH	Effects on human activity change	BUC→HAC	0.61			BUC→HAC	0.61
	Effects on climate change	BUC→CC<br>HAC→CC	0.10<br>0.57	BUC→HAC→CC	0.35	BUC→CC<br>HAC→CC	0.45<br>0.57
	Effects on NPP change	HAC→NPP<br>CC→NPP	0.15<br>0.25	BUC→HAC→NPP<br>BUC→HAC→CC→NPP<br>BUC→CC→NPP<br>HAC→CC→NPP	0.09<br>0.09<br>0.03<br>0.14	BUC→NPP<br>HAC→NPP<br>CC→NPP	**0.21**<br>**0.29**<br>**0.25**
YRD	Effects on human activity change	BUC→HAC	0.25			BUC→HAC	0.25
	Effects on climate change	BUC→CC<br>HAC→CC	0.03<br>-0.67	BUC→HAC→CC	-0.17	BUC→CC<br>HAC→CC	-0.14<br>-0.67
	Effects on NPP change	HAC→NPP<br>CC→NPP	0.10<br>-0.26	BUC→HAC→NPP<br>BUC→HAC→CC→NPP<br>BUC→CC→NPP<br>HAC→CC→NPP	0.03<br>-0.02<br>-0.01<br>0.17	BUC→NPP<br>HAC→NPP<br>CC→NPP	**0.00**<br>**0.27**<br>**-0.26**
PRD	Effects on human activity change	BUC→HAC	-0.71			BUC→HAC	-0.71
	Effects on climate change	BUC→CC<br>HAC→CC	0.27<br>0.01	BUC→HAC→CC	-0.01	BUC→CC<br>HAC→CC	0.26<br>0.01
	Effects on NPP change	HAC→NPP<br>CC→NPP	0.06<br>-0.09	BUC→HAC→NPP<br>BUC→HAC→CC→NPP<br>BUC→CC→NPP<br>HAC→CC→NPP	0.04<br>0.00<br>-0.02<br>0.00	BUC→NPP<br>HAC→NPP<br>CC→NPP	**0.02**<br>**0.06**<br>**-0.09**
YRMR	Effects on human activity change	BUC→HAC	-0.30			BUC→HAC	-0.30
	Effects on climate change	BUC→CC<br>HAC→CC	0.21<br>-0.61	BUC→HAC→CC	-0.18	BUC→CC<br>HAC→CC	-0.03<br>-0.61
	Effects on NPP change	HAC→NPP<br>CC→NPP	0.35<br>-0.22	BUC→HAC→NPP<br>BUC→HAC→CC→NPP<br>BUC→CC→NPP<br>HAC→CC→NPP	-0.10<br>0.04<br>-0.05<br>0.13	BUC→NPP<br>HAC→NPP<br>CC→NPP	**-0.11**<br>**0.48**<br>**-0.22**
CY	Effects on human activity change	BUC→HAC	0.10			BUC→HAC	0.10
	Effects on climate change	BUC→CC<br>HAC→CC	0.14<br>0.13	BUC→HAC→CC	0.01	BUC→CC<br>HAC→CC	0.15<br>0.13

	Effects on NPP change	HAC→NPP<br>CC→NPP	0.14<br>0.75	BUC→HAC→NPP<br>BUC→HAC→CC→NPP<br>BUC→CC→NPP<br>HAC→CC→NPP	0.01<br>0.01<br>0.11<br>0.10	BUC→NPP<br>HAC→NPP<br>CC→NPP	**0.13**<br>**0.24**<br>**0.75**
MSL	Effects on human activity change	BUC→HAC	-0.55			BUC→HAC	-0.55
	Effects on climate change	BUC→CC<br>HAC→CC	0.52<br>-0.63	BUC→HAC→CC	0.35	BUC→CC<br>HAC→CC	0.87<br>-0.63
	Effects on NPP change	HAC→NPP<br>CC→NPP	-0.02<br>-0.15	BUC→HAC→NPP<br>BUC→HAC→CC→NPP<br>BUC→CC→NPP<br>HAC→CC→NPP	0.01<br>-0.05<br>-0.08<br>0.09	BUC→NPP<br>HAC→NPP<br>CC→NPP	**-0.12**<br>**0.07**<br>**-0.15**
